# Pequi Seed Biochar as Pd Nanoparticle Support for Catalytic Hydrogen Evolution from Ammonia Borane

**DOI:** 10.1021/acsomega.5c13606

**Published:** 2026-06-30

**Authors:** Júlia Araújo Lavorato, Marcela de Oliveira Brahim Cortez, Mariele Dalmolin da Silva, Antonio Machado Netto, Lavínia Nunes Louzada, Noemi Cristina Silva de Souza, Leonarde do Nascimento Rodrigues, Renê Chagas da Silva, Luciano de Moura Guimarães, Didier Astruc, Renata Pereira Lopes Moreira

**Affiliations:** † Departament of Chemistry, Universidade Federal de Viçosa (UFV). Av. Peter Henry Rolfs, s/n, Campus Universitário, Viçosa, CEP: 36570-900 MG, Brazil; ‡ Departament of Agricultural Engineering, Universidade Federal de Viçosa (UFV). Av. Peter Henry Rolfs, s/n, Campus Universitário, Viçosa, CEP: 36570-900 MG, Brazil; § Department of Physics, Universidade Federal de Viçosa (UFV), Av. Peter Henry Rolfs, s/n, University Campus, Viçosa, CEP:36570-900 MG, Brazil; ∥ ISM, UMR CNRS N° 5255, University of Bordeaux, Talence Cedex 33405, France

## Abstract

Agribusiness plays a central role in the Brazilian economy, contributing roughly one-quarter of Brazil’s total economic activity. However, it generates approximately 1 million tons of agro-industrial waste per year, often improperly disposed of, leading to environmental impacts. The valorization of this waste for biochar production represents a sustainable alternative. In this work, biochar derived from pequi seeds, an abundant residue from the Brazilian Cerrado, was evaluated as a support for palladium nanoparticles for the synthesis of a catalyst applied to catalytic hydrogen evolution via ammonia borane (NH_3_BH_3_) hydrolysis. The biochar was activated with ZnCl_2_ (BCZ) and produced by pyrolysis, exhibiting a high surface area (1577.8 m^2^ g^–1^) and high porosity, characteristics favorable for metal dispersion. Transmission electron microscopy revealed a homogeneous distribution of nanoparticles, and isotopic analysis in D_2_O indicated a primary kinetic isotope effect (KIE = 6.41), suggesting that water O–H bond cleavage is the rate-determining step of the reaction. The Pd catalyst exhibited a high Turnover Frequency (TOF, 90 min^–1^ at 50 °C) and excellent stability over 20 cycles. These results demonstrate the potential of pequi-derived biochar as a sustainable and low-cost support for clean hydrogen production.

## Graphical abstract

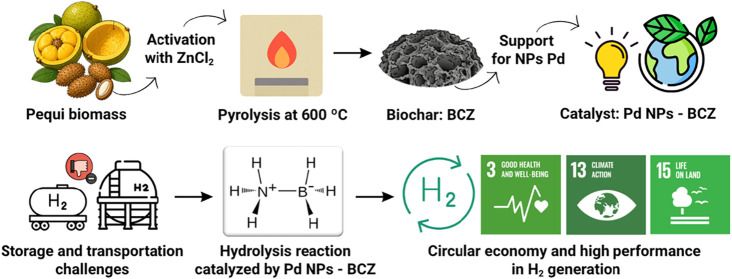

## 1 Introduction

Agribusiness is a strategic sector of the Brazilian economy, playing a central role in strengthening the country’s trade balance. In 2022, agricultural production alone accounted for about 7% of Brazil’s Gross Domestic Product, while the agribusiness sector, including inputs, primary production, and industrial processing, represented approximately 24.8% of the national total.^1^

Despite the high productive performance of agribusiness, it is estimated that about 1 million tons of agro-industrial waste are generated annually in Brazil.^2^ These byproducts, such as leaves, seeds, peels, and bagasse, originate from harvesting and agricultural processing. However, a large portion of this material is inadequately discarded without any valorization, posing a significant environmental concern by contributing to greenhouse gas emissions and the contamination of water bodies and soils.^2^ As a result, practices based on circular economy principles and waste minimization have been increasingly encouraged by international policies promoting sustainable production processes, such as the United Nations Sustainable Development Goals (SDGs) and the 2030 Agenda.

In general, agricultural residues are composed of lignocellulosic materials, mainly cellulose (40–50%), hemicellulose (20–30%), lignin (20–25%), and a small mineral fraction represented by ash (1–5%).^3^ Additionally, they contain several functional groups, such as hydroxyl, carboxyl, carbonyl, and phenolic groups. As low-cost and abundant materials, these residues hold great potential for conversion into value-added products.^4^

Pequi (*Caryocar spp*.), a native fruit of the Brazilian Cerrado, is cultivated mainly in the Southeast, Northeast, and Central-West regions, with Minas Gerais accounting for approximately 74 thousand tons of national production in 2021. The oil extracted from its kernel is used in biodiesel synthesis. However, improper management of the resulting solid residues leads to greenhouse gas emissions, representing an important environmental issue.^5^ In addition, pequi exploitation is largely based on extractive activities carried out by local communities in the Cerrado, highlighting its socio-economic importance. In this context, the valorization of its residues offers an opportunity to strengthen sustainable value chains and support the economic autonomy of these communities.^6^ This perspective is further supported by recent public policies, such as Law 15,089, which establishes guidelines for the sustainable use of pequi, including reforestation initiatives, product certification, and support for extractive communities.^7^ Thus, valorizing this residue as a raw material for biochar production both reduces environmental impacts and promotes the sustainable use of a widely available regional biomass, aligning with SDG 3 (good health and well-being), SDG 13 (climate action), and SDG 15 (life on land).

Among agro-industrial waste management techniques, thermochemical processes, particularly pyrolysis, stand out for their efficiency in biomass conversion. The resulting biochar is a porous material with high surface area and carbon content, characteristics that provide versatility and strong potential for various applications.^8^ One of its relevant applications is as a support in heterogeneous catalysis, due to its structural ability to anchor metallic nanoparticles.^9^ This approach has proven promising for the development of efficient catalysts for hydrogen (H_2_) generation, often resulting in high catalytic activity.

Hydrogen is considered a clean and efficient alternative to fossil fuels and is recognized as one of the key pillars of the global transition toward a low-carbon energy matrix.^10^ It is notable for its high gravimetric energy density (142 kJ g^–1^), approximately three times greater than gasoline on a mass basis.^11^ Moreover, hydrogen combustion releases only water vapor, causing no environmental harm. Nonetheless, its storage remains challenging due to its low density (0.0899 g L^–1^ at 0 °C and 1 atm), extremely low boiling point (−252.87 °C), and high flammability.^12^

Although hydrogen is a promising and widely applicable fuel, its storage and transportation present technical limitations because of its flammability.^13^ For this reason, it is commonly stored in the gaseous phase under high pressure (100–400 bar) or in the liquid phase under cryogenic conditions (approximately −253 °C) to ensure safe handling and transport.^14^ An alternative to overcome these challenges lies in solid-state storage materials, which allows controlled hydrogen release. Among these materials, ammonia borane (NH_3_BH_3_, AB) stands out due to its high hydrogen content (19.6 wt %), low toxicity, and high solubility in common solvents such as water and methanol.^15^ During its hydrolysis, NH_3_BH_3_ releases 3 mol of H_2_ (eq 1).

$${\mathrm{NH}}_{3}{\mathrm{BH}}_{3\left(\mathrm{aq}\right)}+2H_{2}O_{(l)}\to {\mathrm{NH}}_{4}{\mathrm{BO}}_{2\left(\mathrm{aq}\right)}+3H_{2\left(g\right)}$$ 1

Catalysts are required for this H_2_ generation reaction. In this regard, metallic nanoparticles such as Rh, Ru, and Pd are commonly employed.^16^ Among these highly active precious metals, Pd stands out as a preferred option because it combines high catalytic activity with greater natural abundance and lower cost when compared to Rh and Ru, making it a more practical and economically viable choice for scalable applications.^17^ However, their high surface area also promotes nanoparticle agglomeration, which decreases reactivity.^18^ One strategy to overcome this limitation is to deposit the nanoparticles onto support materials, such as biochar, which improves nanoparticle dispersion and stability.

Thus, this work aims to evaluate, for the first time, the use of biochar derived from pequi seeds, an innovative and underexplored biomass source, as a support for palladium nanoparticles (Pd NPs) in the catalytic H_2_-release reaction from NH_3_BH_3_. This unprecedented approach seeks to develop a sustainable and efficient system aligned with circular economy principles and the Sustainable Development Goals, while promoting the valorization of native Cerrado resources and advancing clean and environmentally responsible hydrogen-production technologies.

## 2 Experimental Section

### 2.1 Reagents

In this work, high-purity reagents were used, including: zinc chloride (ZnCl_2_, 97%, Neon, CAS 7646–85–7); hydrochloric acid (HCl, 37%, VETEC, CAS 7647–01–0); sodium borohydride (NaBH_4_, 98,19%, Neon, CAS 16940–66–2); potassium tetrachloropalladate (II) (K_2_PdCl_4_, 98%, Sigma-Aldrich, CAS 10025–98–6); ammonium borane (NH_3_BH_3_, 95%, Sigma-Aldrich, CAS 13774–81–7); and sodium chloride (NaCl, 99%, Synth, CAS 7647–14–5).

### 2.2 Biochar Synthesis (BCZ)

The biomass was collected in the northern region of Minas Gerais. The pequi seeds were obtained by manually removing the pulp with the aid of a knife. The seeds were then dried in an oven for 3 days at 65 °C. Subsequently, they were crushed using a Wiley-type knife mill and sieved through stainless-steel meshes with particle sizes ranging from 40 to 80 mesh. For chemical activation, 10 g of the material was mixed with 30 g of ZnCl_2_ previously dissolved in 50.00 mL of deionized water. The mixture was dried in an oven at 105 °C for 24 h to promote ZnCl_2_ impregnation. After cooling, the impregnated material was transferred to crucibles and subjected to pyrolysis in a muffle furnace, with the temperature increased from 10 to 600 °C over the course of 1 h. The resulting product was cooled, treated with HCl (0.100 mol L^–1^), vacuum-filtered, and washed with deionized water at 80 °C until the filtrate reached neutral pH. Finally, the biochar was dried in an oven for 2 days at 65 °C and stored for further use.

### 2.3 Synthesis of Metallic Nanoparticles (Pd NPs-BCZ)

The metal nanoparticles were synthesized by dispersing 20 mg of biochar in 5.00 mL of Type I water, which was maintained under stirring for 10 min. Subsequently, a Pd solution (72 μmol L^–1^) corresponding to 2 mmol % of metal was added, and the mixture was kept under stirring for an additional 10 min. Then, 5.00 mL of a NaBH_4_ solution (0.100 mol L^–1^) was added to promote the reduction of metal ions, followed by magnetic stirring for another 10 min. After the reaction, the suspension was centrifuged at 4000 rpm for 10 min and washed three times with Type I water.

### 2.4 Material Characterization

The characterization of the functional groups present in the materials was carried out by fourier transform infrared spectroscopy (FTIR) using a BRUKER α II spectrometer equipped with a multiple-reflection attenuated total reflectance (ATR) accessory. The analyses were performed in the spectral range of 400 to 4000 cm^–1^, with 64 scans and a resolution of 4 cm^–1^.

Raman spectroscopy was conducted for the biochar using a Renishaw InVia spectrometer equipped with a 633 nm laser and spectral scanning in the range of 100 to 1300 cm^–1^, in order to evaluate the degree of structural organization of the carbon.

The carbon, hydrogen, nitrogen, and sulfur contents of the biomass and biochar were determined by elemental analysis (CHNS) using a LECO TruSpec Micro analyzer. Cystine was employed as the reference substance, with the combustion tube operating at 1150 °C and the reduction tube at 850 °C. The oxygen content was calculated by difference.

The morphology of the materials was assessed by scanning electron microscopy (SEM) using a JEOL JSM-6010LA microscope operating at an acceleration voltage of 20 kV. The samples were prepared on carbon stubs and coated with gold using a Quorum Q150R S sputter coater.

The surface area of the biochar was determined through nitrogen adsorption–desorption analyses. The isotherms were obtained using a Nova Series 600 analyzer from Anton Paar. The surface area was calculated using the Brunauer–Emmett–Teller (BET) method, and the pore size distribution was derived using the Barrett–Joyner–Halenda (BJH) model. Prior to analysis, the material was degassed at 120 °C for 4 h to remove moisture and residual gases.

X-ray diffraction (XRD) analyses of the biochar and the catalyst were performed using a Bruker D8 Discover diffractometer with Cu Kα radiation (λ = 0.1541 nm), with angular scanning (2θ) in the range of 10° to 90°.

For the ζ-potential analysis, 10 mg of the material were dispersed in 250 mL of NaCl (0.1 mol L^–1^) solution and subjected to magnetic stirring for 24 h. The suspension was then sonicated for 2 h. After this preparation, the pH of the suspension was adjusted to 2, 4, 6, 8, 10, and 12, and the analyses were performed using a Nano ZS Zetasizer from Anton Paar.

The materials were also analyzed by transmission electron microscopy (TEM) using a Tecnai G2–20 FEI SuperTwin microscope operating at 200 kV and equipped with an energy-dispersive X-ray spectroscopy (EDX) system. Prior to analysis, the sample was sonicated for 5 min, and a small volume of the suspension was deposited onto a TEM grid and dried at room temperature.

The catalyst was also analyzed by Inductively Coupled Plasma Optical Emission Spectrometry (ICP-OES, iCAP PRO, Thermo Scientific) for the quantification of Pd, B, and Zn, monitoring the emission lines at 340, 208, and 206 nm, respectively. For sample preparation, 20 mg of catalyst was digested using approximately 20 mL of concentrated sulfuric acid (H_2_SO_4_, 95–97%) and 10 mL of hydrogen peroxide (H_2_O_2_, 130 volumes). The mixture was maintained under continuous stirring and heated at ∼50 °C for 2 h to ensure complete digestion. After cooling, the solution was transferred to a 100.00 mL volumetric flask and diluted to volume with nitric acid solution (1 mmol %). Appropriate dilution steps were applied to keep the sulfur concentration below 2000 mg L^–1^, in accordance with the operational limits of the instrument and to ensure analytical reliability.

### 2.5 Evolution of Hydrogen Gas

The hydrogen gas evolution experiments were carried out by adding Pd NPs-BCZ dispersed in 2.00 mL of Type II water into a Schlenk tube under magnetic stirring. The metallic NPs were reduced using excess NaBH_4_. The tube was sealed with a septum and connected to a water-filled buret. Subsequently, 1.00 mL of an NH_3_BH_3_ solution (0.58 mmol L^–1^) was injected into the system through the septum using a syringe. The volume of hydrogen generated was monitored by measuring the displacement of water in the buret at a given time. The schematic of the system used is shown in Figure S1. Furthermore, the determination of the kinetic parameters of the reaction was performed using the test function F­(C), which involves the comparison of experimental data with integrated rate equations of different orders, according to the methodology described by Bousada et al. (2024)^19^

Different reaction parameters were optimized, including:

I. Metals variation: palladium, ruthenium, rhodium, nickel, and platinum nanoparticles were evaluated under standardized reaction conditions consisting of 2 mmol % metal, 20 mg of BCZ, 0.58 mmol L^–1^ NH_3_BH_3_, and a fixed temperature of 25 °C.
II. Support dosage: BCZ amounts of 10, 20, 30, and 40 mg were tested while maintaining constant conditions of 2 mmol % metal, 0.58 mmol L^–1^ NH_3_BH_3_, and 25 °C.
III. Metal loading: metal doses of 1, 2, 4, and 6 mmol % were assessed under constant conditions of 20 mg BCZ, 0.58 mmol L^–1^ NH_3_BH_3_, and 25 °C.
IV. Reaction temperature variation: reaction temperatures of 20, 25, 30, 40, and 50 °C were investigated while keeping constant conditions of 2 mmol % metal, 20 mg BCZ, and 0.58 mmol L^–1^ NH_3_BH_3_.
V. Kinetic isotope effect (KIE): the procedure was conducted by replacing Type II water with deuterium oxide (D_2_O). After synthesis, the material was washed with isopropyl alcohol and vacuum-dried prior to the addition of D_2_O. The remaining experimental conditions were 2 mmol % catalyst, 20 mg BCZ, 0.58 mmol L^–1^ NH_3_BH_3_, and 25 °C. The KIE value was calculated using eq 2
2 $$\mathrm{KIE}=\frac{k_{H}}{k_{D}}$$
  Where *k*_H_ is the kinetic constant of the event using H_2_O and *k*_D_ is the kinetic constant of the event using D_2_O.
VI. Durability: the H_2_ evolution experiments were conducted under fixed conditions: 2 mmol % catalyst, 20 mg of BCZ, 0.58 mmol L^–1^ NH_3_BH_3_, and 25 °C. After the second cycle, only a fresh NH_3_BH_3_ solution was added, without washing the catalyst. The turnover frequency (TOF) was calculated according to eq 3.
3 $$\mathrm{TOF}=\frac{n_{H_{2}}}{n_{\mathrm{Pd}}\times \Delta t}$$
  Where *n*_H_2__ is the number of moles of H_2_ produced (mmol), *n*_Pd_ is the catalyst moles (mmol) and Δ*t* is the reaction time (min).

The activation energy was obtained using the Arrhenius eq 4.

4 $$\ln K=\ln A-\frac{E_{a}}{\mathrm{RT}}$$

Where *K* is the rate constant of the reaction, *A* is the pre-exponential factor, *E*_a_ is the activation energy, *R* is the universal gas constant and *T* is the reaction temperature.

Representative data are shown for each reaction condition, in line with standard practice in heterogeneous catalysis. All experiments were conducted under identical, strictly controlled conditions and repeated on separate days to confirm the reproducibility and reliability of the observed trends.

## 3 Results and Discussion

### 3.1 Characterization of Materials

Figure 1(a) shows the FTIR spectra of the pequi biomass, the biochar obtained by pyrolysis, and the catalyst (Pd NPs–BCZ). The biomass shows a broad band centered around 3300 cm^–1^, assigned to the O–H stretching vibrations of hydroxyl groups, associated with adsorbed water, carboxylic acids, and alcohols. In the 3000–2750 cm^–1^ region, bands corresponding to the C–H stretching of sp^3^-hybridized carbons, mainly *ν*_as_(CH_2_) and *ν*_s_(CH_3_), are observed, which are characteristic of aliphatic compounds.^20^ The band at approximately 1750 cm^–1^ is associated with C=O stretching, ν­(C=O), while the region between 1150 and 1000 cm^–1^ corresponds to C–O/C–O–C vibrations, typical of ketones, aldehydes, lactones, and carboxylic groups.^21^ After pyrolysis, the biochar spectrum shows a reduction in the intensity of O–H and aliphatic C–H bands (3000–2750 cm^–1^), indicating thermal degradation of oxygenated groups and the loss of volatile and lipidic components. This behavior is associated with the decrease in the O/C and H/C molar ratios (Table 1), resulting from dehydration and decarboxylation reactions. In contrast, the appearance of a new band at ∼1600 cm^–1^, attributed to aromatic C=C stretching, ν­(C=C), suggests an increase in aromaticity and structural condensation of the carbon matrix, in agreement with the literature.^22^ For the Pd NPs–BCZ catalyst, the spectrum retains the main features of the biochar, indicating that metal incorporation does not significantly alter the chemical framework or surface functional groups of the support, which is consistent with the low Pd loading.^23^

**Figure 1:**
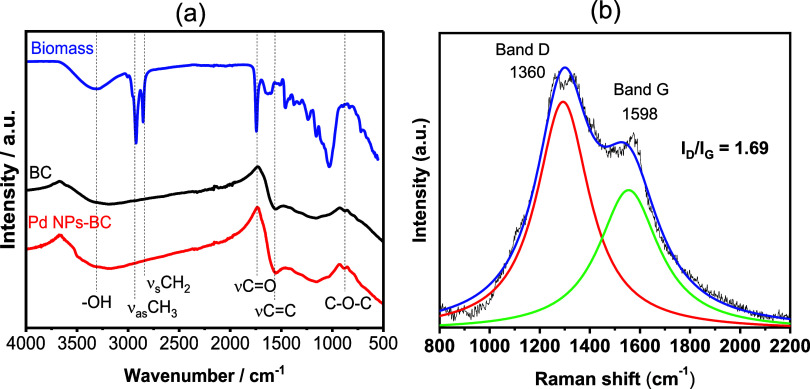
FTIR of (a) pequi biomass, biochar (BCZ) and catalyst (Pd NPs-BCZ) (b) Raman spectroscopy of Pd NPs-BCZ.

**Table 1:** Elemental Analysis (CHNS) of Biomass and Biochar (BCZ)

material	C(% m/m)	H(% m/m)	N (%m/m)	S(% m/m)	O(% m/m)	H/C	O/C
Biomass	52.19	6.80	1.36	1.64	38.02	1.55	0.55
BCZ	66.41	2.98	2.69	<LQ[Table-fn t1fn1]	30.91	0.53	0.35

b LQ: Limit of quantification.

The composition of the biochar support (BCZ) was characterized by Raman spectroscopy, and the corresponding spectrum is shown in Figure S2. Two prominent peaks are observed at approximately 1360 and 1598 cm^–1^, corresponding to the D and G bands, respectively. The D band is associated with structural defects and disorder in the carbon lattice, whereas the G band is related to the stretching vibrations of sp^2^ carbon atoms, characteristic of graphitic domains.^24^ The intensity ratio between these bands (*I*_D_/*I*_G_ = 0.93) indicates a moderate degree of structural disorder and the coexistence of both graphitic and amorphous domains within the carbon matrix.^25,26^ These results are consistent with values reported for biochars with high structural stability and a predominance of graphitic carbon.^27^ After the deposition of Pd nanoparticles (Pd NPs), the *I*_D_/*I*_G_ ratio increased to 1.69, as shown in Figure 1(b). This change is associated with the overall catalyst preparation process, including nanoparticle formation, metal nucleation, anchoring onto the carbon surface, and the reductive environment provided by NaBH_4_ during synthesis.^28^ These conditions may induce local distortions in the sp^2^ domains, increase sp^3^ hybridization, and generate vacancies and edge defects. These changes enhance the structural disorder detected by the D band. Therefore, the higher *I*_D_/*I*_G_ ratio reflects an increase in structural disorder of the carbon matrix after catalyst formation, resulting from the combined effects of the synthesis process rather than a single isolated contribution such as Pd deposition alone.

The results of the elemental analysis (CHNS) of the biomass and biochar are presented in Table 1. Both materials are predominantly composed of carbon, followed by oxygen, hydrogen, and nitrogen.^29^ A marked increase in carbon content (from 52.19 to 66.41%) and a significant decrease in hydrogen and oxygen contents was observed, reflecting the chemical transformations occurring during the pyrolysis process. This behavior is associated with the elimination of volatile compounds and the removal of oxygenated groups, promoting carbon concentration and the formation of more aromatic and thermally stable structures.^30^ The atomic ratios H/C and O/C decreased from 1.55 to 0.53 and from 0.55 to 0.35, respectively, indicating the occurrence of dehydrogenation, deoxygenation, and aromatic condensation reactions typical of carbonization processes.^20^ The reduction in the H/C ratio reflects the loss of aliphatic components, whereas the decrease in the O/C ratio is associated with decarboxylation and dehydration. These values suggest a more condensed and stable carbonaceous structure with a lower content of reactive functional groups, features that are desirable for use as a catalytic support and as an adsorbent in environmental systems.

The morphology of the obtained materials was analyzed by SEM, as shown in Figure 2. The biomass (Figure 2a) exhibits a fibrous and relatively compact structure, typical of lignocellulosic materials, with well-defined bundles and cell walls. After the pyrolysis process (Figure 2b), an intensely fragmented surface with no defined morphology is observed, composed of irregularly shaped particles of various sizes, indicating the thermal degradation of organic constituents and the partial collapse of the original structure. After the deposition of palladium nanoparticles (Figure 2c), agglomerates can be observed distributed across the biochar surface, a behavior also reported by Walbrück et al. (2019)^31^ The material obtained by impregnation followed by pyrolysis exhibits structural features favorable for the anchoring and dispersion of metallic nanoparticles, an essential aspect for the use of biochar as a catalytic support.^32^

**Figure 2:**
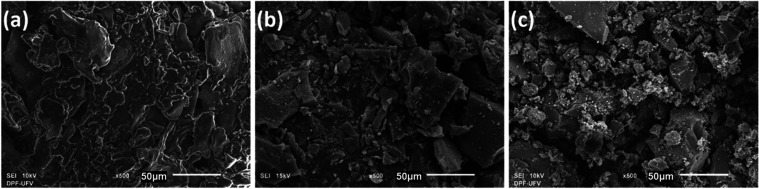
Scanning Electron Microscopy (SEM) for (a) biomass, (b) biochar and (c) Pd NPs-BCZ.

The N_2_ adsorption–desorption results for the prepared materials are shown in Figure S3. The isotherms reveal substantial changes in the porous structure after chemical activation and subsequent Pd deposition. The nonactivated BC exhibits a specific surface area of 8.79 m^2^ g^–1^, a total pore volume of 0.0045 cm^3^ g^–1^, and an average pore diameter of 34.32 nm, consistent with a largely nonporous or macroporous material. Its type II isotherm (Figure S3a) confirms this behavior and is typical of carbons with poorly developed porosity. The presence of irreversible adsorption at higher relative pressures suggests inelastic distortion of less rigid biomass domains that remain in the carbonaceous matrix.^33^ Chemical activation with ZnCl_2_ dramatically enhances the textural properties of the material. The BCZ sample presents a specific surface area of 1577.80 m^2^ g^–1^, a total pore volume of 0.79 cm^3^ g^–1^, and an average pore diameter of 3.42 nm, indicative of a well-developed mesoporous network. Its type IV isotherm with an H4 hysteresis loop (Figure S3b) reflects the coexistence of micropores and narrow slit-shaped mesopores, generating a heterogeneous micro–mesoporous framework.^34,35^ These values surpass those reported by Zhang et al. (2024)^36^ for ZnCl_2_-activated biochars derived from *Spartina alterniflora*, whose surface areas ranged from 909.79 to 1062.14 m^2^ g^–1^ and pore volumes from 0.17 to 0.40 cm^3^ g^–1^, depending on the precursor/activator ratio. This comparison highlights the high efficiency of the activation procedure used in the present study. The Pd-loaded sample (Pd NPs-BCZ) also exhibits a type IV isotherm; however, it displays an H3 hysteresis loop, which is typically associated with open slit-like mesopores and reflects a reduced contribution of micropores. The incorporation of Pd nanoparticles leads to a decrease in textural parameters, resulting in a specific surface area of 707.74 m^2^ g^–1^, a pore volume of 0.16 cm^3^ g^–1^, and an average pore diameter of 3.45 nm (Figure S3c). This change may be associated with partial blocking of micro- and mesopores by the metal nanoparticles.^37^ Additionally, wet impregnation and reduction steps may induce structural rearrangements in the porous carbon framework, and metal impregnation may modify the pore structure and block pore openings, thereby decreasing the accessible surface area.^38^ For instance, Butenko observed a reduction in BET surface area from 1310 to 438 m^2^ g^–1^ following the formation of Pd nanoclusters.^39^ This behavior was attributed to the overlap between the pore size distribution of the carbon support and the size of the Pd nanoclusters, indicating that the nanoparticles were formed within the pores. Similarly, in the present work, the decrease in surface area suggests that the deposition of Pd nanoparticles may partially block micro- and mesopores, leading to a reduction in the accessible surface area. The shift in hysteresis loop type thus provides further evidence of structural modification of the porous network following Pd deposition.

The combination of high surface area and interconnected mesoporous structure makes BC an efficient support for the dispersion of metallic nanoparticles, providing more anchoring sites and minimizing the agglomeration of active particles. This structural configuration favors diffusion and contact between reactants and catalytic sites, resulting in improved performance in heterogeneous catalytic systems aimed at hydrogen production.^32^

The X-ray diffraction (XRD) pattern of the biochar (BC) and catalyst (Pd NPs-BCZ) are shown in Figure S4­(a,b). A broad band is observed in the 2θ range between 20° and 30°, characteristic of amorphous carbonaceous materials, a typical feature of biochars derived from lignocellulosic biomass and other carbon materials with predominantly disordered structures.^40^ The absence of well-defined crystalline peaks confirms the low crystallinity of the material, which is attributed to the thermal decomposition of the structural components of the biomass, mainly cellulose, hemicellulose, and lignin, during the pyrolysis process. The reduction in crystallinity results from the breakdown of inter- and intramolecular hydrogen bonds in lignocellulosic fibers, disrupting the semicrystalline regions of cellulose and leading to the formation of an amorphous carbon matrix.^41^ This disordered structure is favorable for catalytic applications, as the structural defects and residual oxygen-containing functional groups on the carbon surface act as active sites for the anchoring and uniform dispersion of metallic nanoparticles.^42^ After Pd deposition, no distinct diffraction peaks corresponding to Pd or its crystalline phases could be detected. This is attributed to the low metal loading relative to the carbon support, as well as the high dispersion and small size of the Pd NPs, which result in diffraction signals below the detection limit of conventional XRD.

The ζ-potential evaluation of the BCZ is presented in Figure S5. The measured values were negative across the entire pH range analyzed (2–12), indicating that the surface of the material remains negatively charged throughout this interval. The further decrease in ζ-potential at more alkaline pH values is associated with the increased density of negative charges due to the deprotonation of oxygen-containing functional groups, such as surface carboxyl and hydroxyl groups. This feature provides greater colloidal stability to the material in aqueous media, as electrostatic repulsion between particles reduces the tendency for agglomeration and, consequently, minimizes the loss of active surface area.^43^ The negative surface charge of the BCZ favors electrostatic interactions with cationic metal species, promoting the anchoring and homogeneous dispersion of nanoparticles. This characteristic also contributes to improved electron transfer between the support and the metallic particles, thereby enhancing both the catalytic activity and the stability of the system.^44^

The Pd NPs–BCZ catalyst was characterized by TEM, as shown in Figure 3. In Figure 3(a), the graphene-like sheets of the biochar matrix are clearly visible, providing a structured and conductive surface for nanoparticle anchoring. The TEM images confirm that the palladium nanoparticles are uniformly dispersed across the support (Figure 3(b)). The particle-size histogram (Figure 3c) shows an average diameter of 4.29 ± 1.43 nm. This narrow size distribution is consistent with the morphology observed in Figure 3(d), where the well-distributed nanoparticle sizes can be clearly seen. A similar result was reported in Ma et al. (2024)^45^ in which Pd nanoparticles supported on CuFe_2_O_4_ were employed in the dehydrogenation of NH_3_BH_3_, exhibiting an average particle size of approximately 3.5 nm. The EDS results are shown in Figure S6, revealing the presence of carbon (77.9%) and oxygen (6.5%), both characteristic of the BCZ, as well as a Pd signal (10.6%), which confirms the successful incorporation of the metal into the material. The detection of Zn is attributed to the activation process.

**Figure 3:**
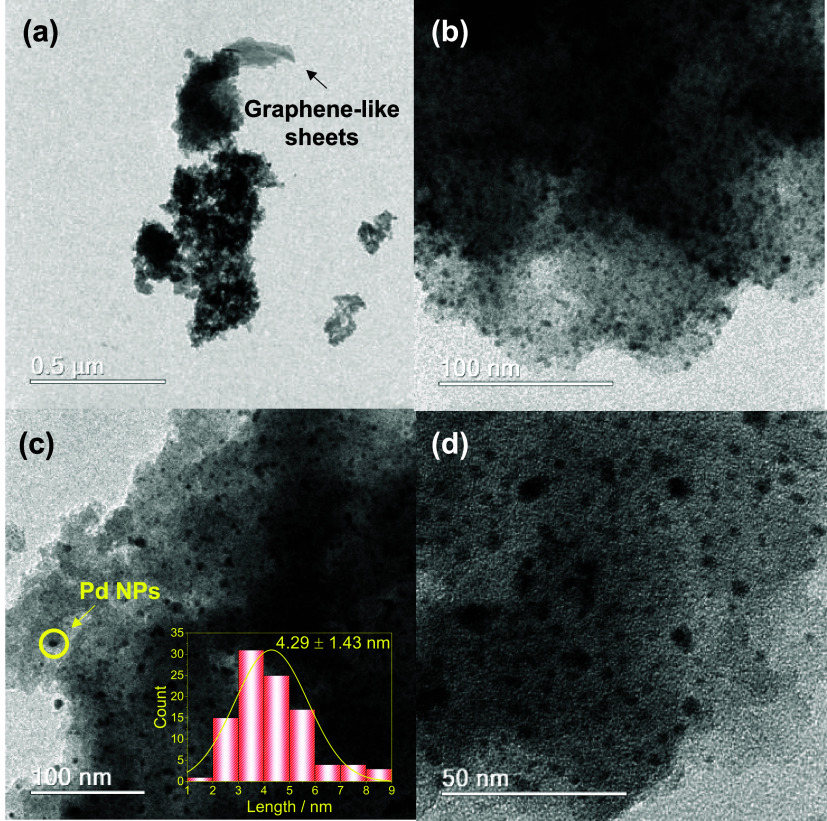
Transmission electron microscopy (TEM) of Pd NPs-BCZ: (a) Highlight of the graphene-like sheets; (b) Highlight of the Pd NPs dispersion; (c) Particle-size histogram; (d) Highlight of the Pd NPs.

From the Selected Area Electron Diffraction (SAED) pattern (Figure S7), three diffraction rings can be observed. The measured ring spacings at 2.25, 1.92, and 1.18 Å correspond to the (111), (200), and (311) planes of Pd, confirming the crystalline nature of the nanoparticles. These results are consistent with the JCPDS card 46–1043 for palladium. Similar findings were reported by Choudhary et al. (2024)^46^ for a composite composed of graphene oxide and palladium nanoparticles.

### 3.2 H_2_ Evolution

Transition metal-based nanomaterials usually serve as catalysts in the hydrolysis of NH_3_BH_3_, with noble metals typically achieving higher hydrogen generation rates than non-noble ones.^47^ Some studies have shown that combining different non-noble metals can enhance catalytic performance. For example, copper-based bimetallic systems have demonstrated improved activity in hydrogen production from chemical hydrogen storage materials.^48^ Then, BCZ was used as a support for the metallic nanoparticles, forming the catalysts applied in the hydrogen evolution reaction. Different metals were evaluated with respect to their anchoring behavior and the catalytic performance of each system, as shown in Figure 4a,b. Among the evaluated metals, palladium exhibited the highest performance, as demonstrated by the highest TOF value of 79.05 min^–1^ (Figure 4a) and the fastest reaction kinetics (Figure 4b), resulting in superior catalytic activity for the NH_3_BH_3_ hydrolysis reaction. A similar result was reported by Sun et al. (2023)^49^ who employed the Pd/Ti_3_C_2_ catalyst in the hydrolysis of NH_3_BH_3_ for H_2_ production, obtaining a TOF of approximately 59 min^–1^.^50^ reported a TOF of 125 min^–1^ for the hydrolysis of NH_3_BH_3_ using Pd nanoparticles immobilized on mesoporous carbon nitride. However, their system required a relatively high metal loading (3 mmol % Pd), and the support employed was not derived from agro-industrial waste, in contrast to the sustainable carbon matrix explored in the present work.

**Figure 4:**
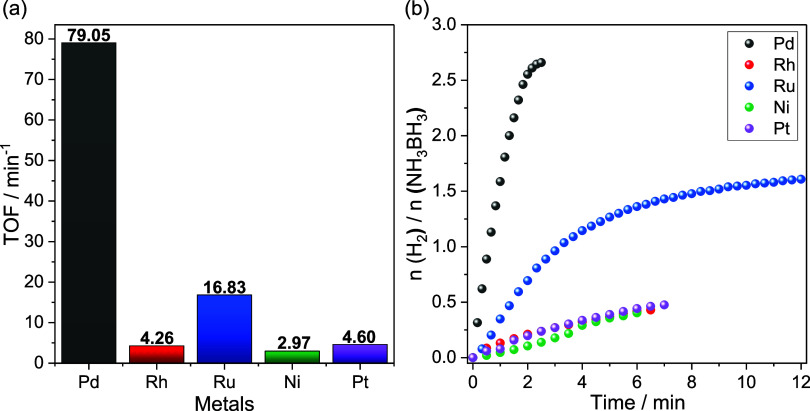
Evaluation of metal variants for hydrogen evolution from NH_3_BH_3_. (a) TOF values; (b) kinetic relationships. Reaction conditions: 2 mmol % metal, 20 mg BCZ, 0.58 mmol L^–1^ of NH_3_BH_3_ and 298.15 K.

The hydrogen evolution experiments were also conducted under different conditions using only Pd NPs without BCZ, using Pd NPs-BCZ, using Pd NPs supported on nonactivated biochar (Pd NPs-BC), and using only the BCZ (Figure 5). As shown in Figure 5(a,b), neither the isolated BCZ nor the Pd NPs-BC generated hydrogen under the evaluated conditions. Furthermore, the Zn content in the material was evaluated by ICP analysis (Table S1). To investigate its possible contribution to the catalytic activity, control experiments using ZnCl_2_ and ZnCl_2_ combined with Pd NPs were performed, and no H_2_ generation was observed (Figure S8). These results indicate that ZnCl_2_ does not directly contribute to the catalytic hydrogen evolution reaction. Instead, its role is associated with the activation of the carbon matrix, promoting the development of surface area and porosity favorable for Pd nanoparticle immobilization, as confirmed by the N_2_ adsorption–desorption analysis.

**Figure 5:**
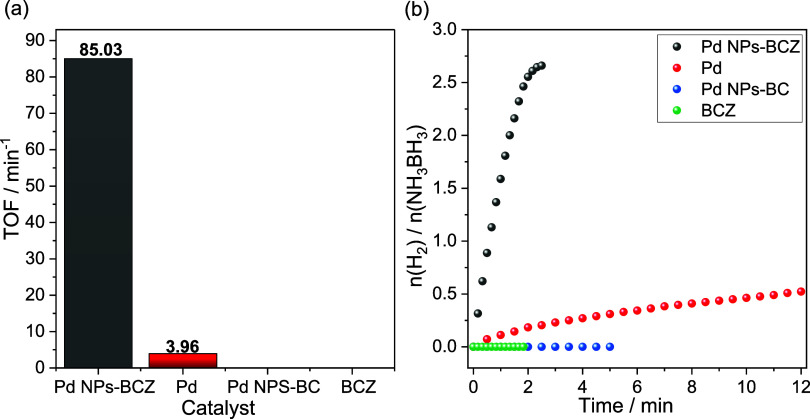
Evaluation of catalyst variants for hydrogen evolution from NH_3_BH_3_. (a) TOF values; (b) kinetic relationships. Reaction conditions: 2 mmol % metal, 20 mg BC or BCZ, 0.58 mmol L^–1^ of NH_3_BH_3_ and 298.15 K.

Another parameter evaluated was the support dosage, as it has a significant impact on catalytic performance during H_2_ evolution. As shown in Figure 6(a, b), increasing the catalyst dosage from 10 to 20 mg led to a marked improvement in hydrogen generation, not only because more active sites became available but also due to the better dispersion of the catalytic material throughout the reaction medium, enhancing the contact between NH_3_BH_3_ and the Pd active sites. However, when the dosage was further increased to 30 and 40 mg, a decline in hydrogen evolution was observed, mainly due to the excess of solid in the medium, which increases mass-transfer resistance and physically limits the accessibility of active sites, reducing the overall catalytic efficiency. Therefore, 20 mg of BCZ was selected and maintained as the reference support dosage for the catalytic evaluation.

**Figure 6:**
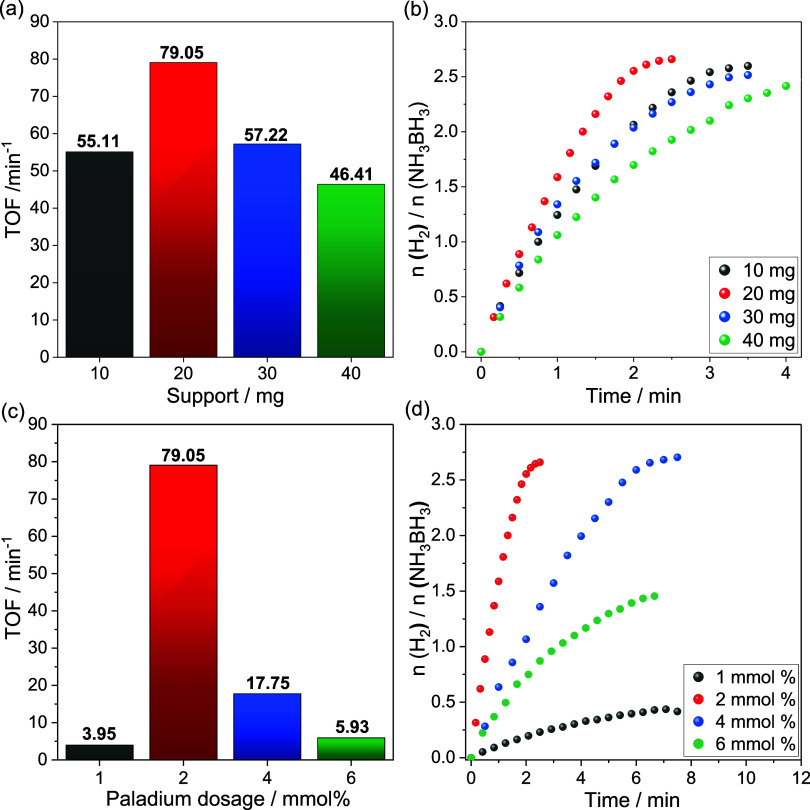
(a, b) Evaluation of BCZ dosage* (c, d) Evaluation of Pd NPs loading** on hydrogen evolution from NH_3_BH_3_ mediated by Pd NPs-BCZ Reaction conditions: 2 mmol % of Pd NPs, 20 mg of BCZ, NH_3_BH_3_ (0.58 mmol L^–1^) and 298.15 K.

The effect of Pd loading was evaluated, and the results are presented in Figure 6(c,d). The catalyst dosage showed a strong influence on the system’s performance, with the 2 mmol % Pd loading achieving the highest TOF and clearly outperforming the other evaluated dosages. For higher metal loadings, a marked decrease in catalytic activity was observed. This decline may be related to the excessive amount of metal on the support, which can limit the effective exposure of active sites and reduce the overall catalytic efficiency.^16^ A similar trend was reported by Zhang et al. (2025)^51^ who employed the Pd_0.5_%Co_35_%/B-HNT catalyst and observed that increasing the catalyst amount initially enhanced the AB hydrolysis rate; however, beyond a certain threshold, the yield decreased. Therefore, at 2 mmol % of Pd, there is a greater availability of active sites and adequate metal dispersion, which enhances the reaction efficiency.

The effect of temperature on the hydrogen evolution reaction with NH_3_BH_3_ is presented in Figure 7. As shown in Figure 7(a,b), increasing the temperature enhanced the TOF, reaching 158.6 min^–1^ at 323.15 K. These results clearly demonstrate that hydrogen generation is strongly temperature dependent.^52^ observed a similar trend, indicating that increasing the temperature enhances the H_2_ generation rate, as higher thermal conditions promote the hydrolysis reaction of NH_3_BH_3_. The acceleration of the reaction rate is attributed to the higher thermal energy of the system, which improves mass transfer and increases the frequency of effective collisions between the reacting species.^53^ In addition to the increase in reaction rate, a decrease in the H_2_ generation efficiency is observed at higher temperatures. At 323.15 K, the efficiency drops significantly compared to lower temperatures (e.g., 293.15 K). This behavior is likely related to the saturation of catalytic sites: as the reaction rate increases, H_2_ is produced more rapidly, leading to higher local H_2_ concentration around the catalyst. This accumulated H_2_ can block active sites, hindering substrate access and reducing overall efficiency.^54^ From the temperature-dependent results, the activation energy (*E*_a_) of the process was determined to be 38.81 kJ mol^–1^, based on the test function and the Arrhenius model (Figure 8(c,d)).

**Figure 7:**
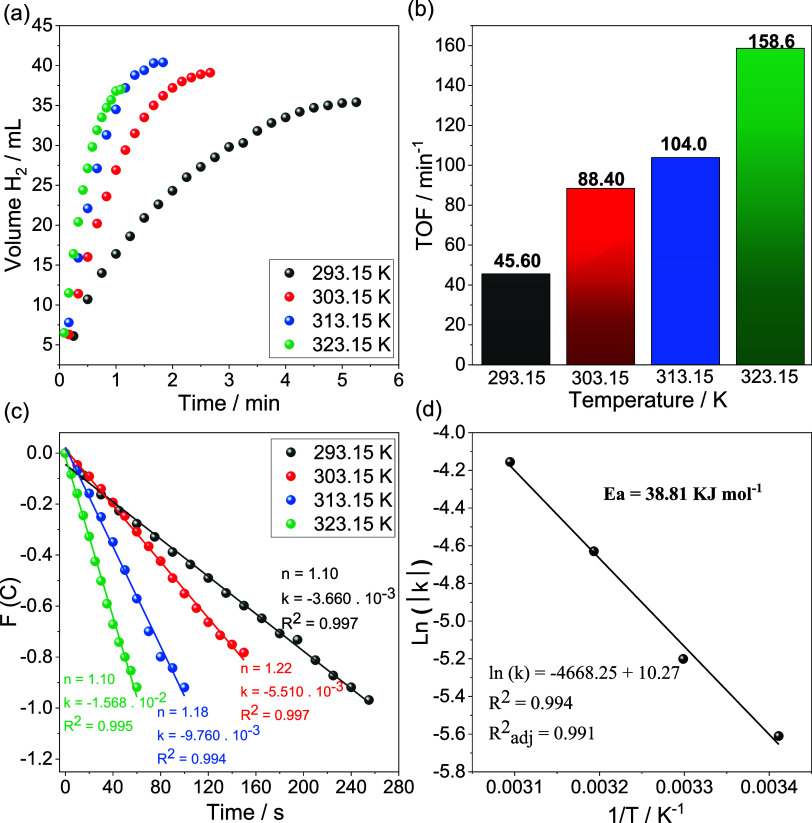
Evaluation of the temperature effect on hydrogen evolution from NH_3_BH_3_ mediated by Pd NPs-BCZ (a) hydrogen production as a function of time, (b) TOF, (c) kinetic relationships, and (d) activation energy determination using the Arrhenius. Reaction conditions: Pd NPs: 2 mmol %, 20 mg of BCZ, NH_3_BH_3_ (0.58 mmol L^–1^).

**Figure 8:**
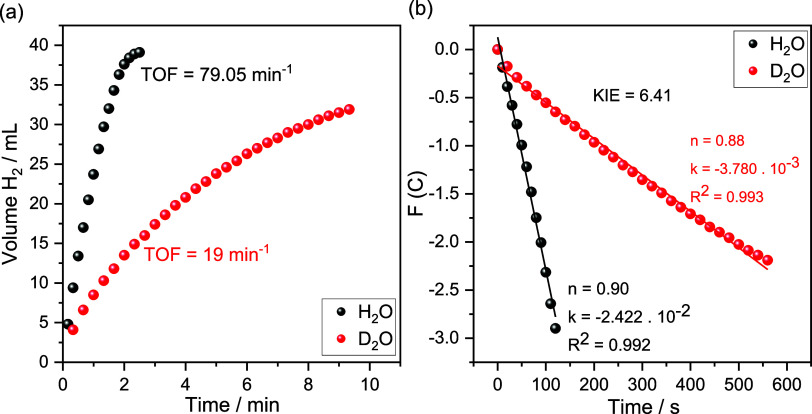
(a) Production of hydrogen and TOF using H_2_O and D_2_O. (b) Determination of Kinetic isotope effect (KIE). Reaction conditions: 2 mmol %, 20 mg of BCZ, 0.58 mmol L^–1^ of NH_3_BH_3_ and 298.15 K.

The linear fit exhibited excellent agreement, as indicated by the high correlation coefficient (*R* ^2^ = 0.994). Comparable values have been reported in the literature.^55^ obtained an *E*_a_ of 36.9 kJ mol^–1^ for H_2_ generation from NH_3_BH_3_ using a palladium–cobalt alloy catalyst. In contrast,^56^ reported a higher *E*_a_ value (52.3 kJ mol^–1^) when employing nickel, palladium, and molybdenum nanoparticles reduced by cesium borohydride.

The obtained KIE value was 6.41 (Figure 8), indicating that the rate-limiting step of the reaction involves the cleavage of the O–H bond in water, since substitution by deuterium (O–D) leads to a significant decrease in the reaction rate.^57^ A similar result was found by Yang et al. (2020)^58^ KIE values in the range of 2 to 7 indicate that the rate-limiting step involves a bond to the labeled atom, whereas values between 0.7 and 1.5 suggest that this bond is neither formed nor broken during the slow step.^16^ Thus, the data reinforces that the activation of the water molecule is a crucial step in the catalytic mechanism, being directly responsible for controlling the reaction rate.

Finally, the catalytic efficiency was evaluated over 20 successive reuse cycles, as shown in Figure 9. Based on the results, it can be observed that the dehydrogenation efficiency of NH_3_BH_3_ for H_2_ production remained high throughout the cycles, with efficiency values close to 100%. These results indicate that the catalyst retained a high catalytic activity even after multiple reaction cycles, with stability and dispersion of the Pd NPs shown in Figure S9. This performance demonstrates that the palladium-based catalyst exhibits high structural stability, a relevant characteristic for hydrogen generation.^59^

**Figure 9:**
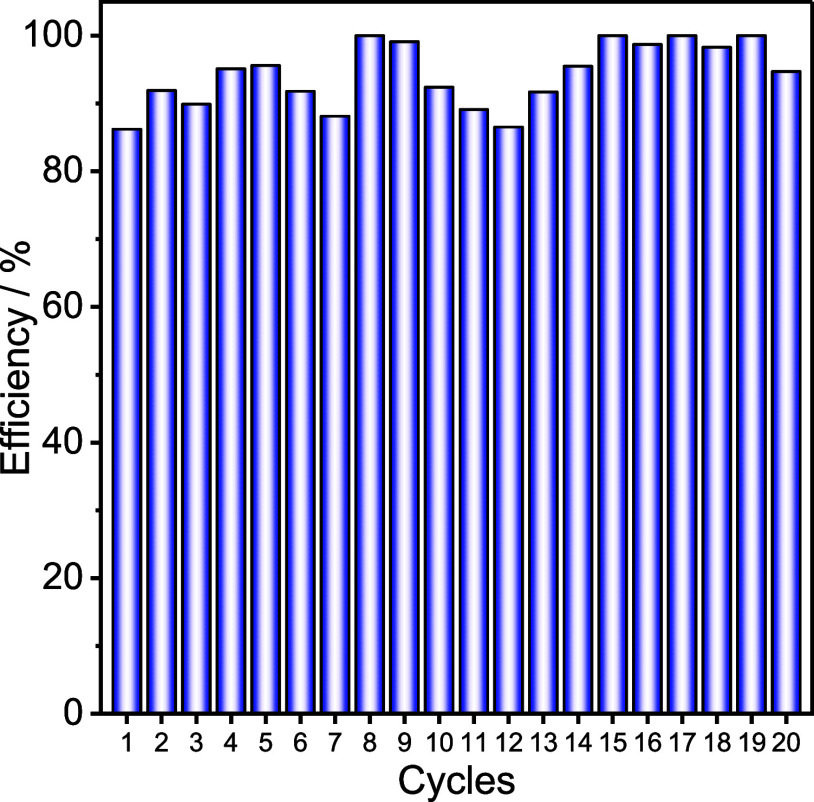
Catalyst durability. Reaction conditions: 2 mmol %, 20 mg de BCZ, 0.58 mmol L^–1^ of NH_3_BH_3_ and 293.15 K.

The mass of Pd and B present in the catalyst was determined by ICP analysis, as summarized in Table S1. Measurements were performed before and after hydrogen evolution from NH_3_BH_3_. The ICP results indicate that the effective Pd loading corresponds to approximately 88% of the nominal value (2 mmol % relative to 0.58 mmol of NH_3_BH_3_), reflecting the actual Pd content incorporated into the biochar-supported catalyst. This result is associated with the incorporation efficiency during the impregnation and washing steps involved in the catalyst preparation. After hydrogen evolution, the Pd content remains essentially unchanged, with variations within experimental uncertainty (∼1%), indicating good stability of the active phase under reaction conditions. Boron was also detected by ICP analysis. Considering the use of excess sodium borohydride for the reduction of Pd from potassium tetrachloropalladate (II), residual boron species may remain associated with the material. After hydrogen evolution, a 32-fold increase in boron content is observed, consistent with the formation of ammonium metaborate as a byproduct of NH_3_BH_3_ dehydrogenation. Therefore, considering the experimentally determined Pd loading by ICP, the turnover frequency (TOF) was calculated as 89.90 min^–1^ using 20 mg of BCZ at room temperature for hydrogen evolution from NH_3_BH_3_.

The data obtained in this work were compared with those reported in the literature, and the performance of the materials is summarized in Table 2.

**Table 2:** Turnover Frequency (TOF), Activation Energy (*E*_a_), and Number of Cycles for Ammonia Borane Dehydrogenation at Room Temperature from Pd-Containing Catalysts

catalyst	TOF (min^–1^)	*E*_a_ (kJ mol^–1^)	cycles	reference
**Palladium anchored on N-doped porous carbon nanosheets (NPC), synthesized via the low-temperature regulation (LTR) method, (Pd/NPC-LTR)**	348.00	41.10	10^a^	^60^
**Ni**_**x**_ **Pd**_**y**_ **Mo**_**z**_ **nanoparticle catalyst prepared via in situ reduction using Cs[closo-B6H7] as the reductant (Ni**_**0.3**_ **Pd**_**0.7**_ **Mo**_**0.2**_ **)**	252.70	52.30	5^b^	^61^
**Amine-terminated CuMnO**_**2**_ **(CuMnO**_**2**_ **-NH**_**2**_ **) supported palladium nanoparticles, (Pd/CuMnO**_**2**_ **-NH**_**2**_ **)**	146.68	84.68	10^b^	^62^
**Palladium supported on mesoporous carbon nitride (MCN), (Pd/MCN)**	125.00	57.00	5^b^	^50^
**Palladium supported on porous carbon nanosheets (IPCNs), (Pd/IPCNs)**	122.80	29.10	4^a^	^63^
**Palladium(0) nanoparticles, supported on carbon-coated magnetic iron particles, (Pd** ^**0**^ **/C–Fe NPs)**	29.00	-	5^a^	^64^
**Pd nanoparticles anchoring to core–shell Fe**_**3**_ **O**_**4**_ **@SiO**_**2**_ **with porous carbon (PC), (Fe**_**3**_ **O**_**4**_ **@SiO**_**2**_ **-PC)**	47.30	28.40	10^a^	^65^
**Pd-doped Cu nanoparticles confined by ZIF-67@ZIF-8** ^c^ **composite,(CuPd**_**0.01**_ **@ZIF-67@ZIF-8)**	30.15	38.78	5^b^	^66^
**Periodic mesoporous organosilicas (PMOs) as support for palladium, (Pd@PMOs)**	30.08	34.90	5^a^	^67^
**Palladium(0) nanoparticles supported on nanoceria (Pd** ^**0**^ **/CeO**_**2**_ **)**	29.00	68.00	5^a^e 5^b^	^68^
**Pequi Seed Biochar as Pd Nanoparticle Support (Pd NPs-BCZ)**	89.90	38.81	20^b^	this work

a Reuse assay (use of the material followed by washing and reapplication in the reaction).

b Durability assay (adding the same dosage of ammonium borane at the end of each evolution).

c ZIF-8: Zeolitic Imidazolate Framework-8 and ZIF-67: Zeolitic Imidazolate Framework-67.

According to Wang et al. (2020), a plausible mechanism for the catalytic hydrogen evolution from NH_3_BH_3_ over Pd NPs-BCZ can be proposed.^13^ Initially, NH_3_BH_3_ adsorbs onto the surface of Pd nanoparticles through interactions involving B–H bonds. This adsorption facilitates the polarization and subsequent cleavage of B–H bonds, generating surface-bound hydrogen intermediates. Simultaneously, water molecules are activated at the Pd/support interface, where the presence of biochar promotes efficient dispersion of Pd and may assist in charge redistribution. The kinetic isotope effect (KIE = 6.41) indicates that O–H bond cleavage in water contributes to the rate-determining step, suggesting that water activation is closely coupled with hydrogen release from NH_3_BH_3_. The hydrogen atoms generated from both NH_3_BH_3_ and water are subsequently recombined on Pd active sites to form H_2_, which is released from the catalyst surface. Biochar support plays a crucial role in stabilizing Pd nanoparticles and maintaining high dispersion, thereby maximizing the number of accessible active sites and enhancing catalytic efficiency. Overall, the reaction proceeds through a cooperative pathway involving Pd nanoparticles as the active centers and the biochar support as a structural and electronic promoter, enabling efficient hydrogen evolution under mild conditions.

## Conclusion

This study demonstrated that the biochar obtained from pequi seed and activated with ZnCl_2_ is an efficient support for the anchoring of palladium nanoparticles. Furthermore, the results highlight the fact that this catalyst exhibits high catalytic activity in the hydrolysis of NH_3_BH_3_ for H_2_ production, achieving a TOF of 89.90 min^–1^ (2 mmol %, 20 mg de BCZ, 0.58 mmol L^–1^ of NH_3_BH_3_ and 293.15 K), with an *E*_a_ of 38.81 kJ mol^–1^ during 20 cycles. Isotopic analysis in D_2_O indicated a primary kinetic isotope effect (KIE = 6.41), suggesting that water O–H bond cleavage is the rate-determining step of the reaction. Thus, the development of this catalyst combines sustainability and high performance, valorizing an abundant regional biomass as support and proving to be a promising alternative for clean H_2_ production. This approach is aligned with the principles of circular economy and sustainable development goals, as it converts agricultural waste into a high-value material while minimizing environmental impacts.

## Supplementary Material



## Notes

Experimental setup used in the reactions: (1) thermostatic bath for temperature control; (2) Schlenk tube; (3) rubber septum for sealing; (4) thermometer; (5) connection tubing; (6) buret; and (7) system open to the atmosphere (Figure S1); Raman spectroscopy of biochar (BCZ) (Figure S2); N_2_ adsorption–desorption Isotherm of (a) biochar without ZnCl_2_, (b) biochar with ZnCl_2_ (BCZ) and (c) metal nanoparticles supported in biochar (Pd NPs-BCZ) (Figure S3); X-ray Diffraction (XRD) of (a) biochar (BC) and (b) Pd NPs-BCZ catalyst (Figure S4); ζ-Potential of biochar (BCZ) (Figure S5); energy dispersive spectroscopy (EDS) spectrum for Pd NPs-BCZ (Figure S6); selected-area electron diffraction (SAED) pattern of Pd NPs–BCZ (Figure S7); evaluation of catalyst variants for hydrogen evolution from NH_3_BH_3_ of (a) TOF values and (b) kinetic relationships. Reaction conditions: 2 mmol % metal, 20 mg BCZ, 0.58 mmol L^–1^ of NH_3_BH_3_ and 293.15 K (Figure S8); transmission electron microscopy (TEM) of Pd NPs-BCZ after 20 successive reuse cycles (Figure S9); ICP analysis of Pd, B, and Zn contents in the materials before and after H_2_ evolution experiments (Table S1) (PDF)

## References

[1] da
Silva Medina G., da Costa R. B. (2023). Building Agro-Industrial Capabilities
in the Sugarcane Supply Chain in Brazil. Logistics.

[2] Visser E. D., Seroka N. S., Khotseng L. (2024). Recent Advances in
Biochar: Synthesis
Techniques, Properties, Applications, and Hydrogen Production. Processes.

[3] Lopes R. P., Astruc D. (2021). Biochar as a support
for nanocatalysts and other reagents:
Recent advances and applications. Coord. Chem.
Rev..

[4] Elsheref M., Ahmed A., Elmelegy E., Tarr M. A., Hammad W., Darweesh M. A. (2024). Adsorptive potential
of apricot (Prunus Armeniaca)
stone in the removal of Cr (VI) and Fe (II) ions from Aquatic Systems:
Kinetic and isothermal investigations. J. Hazard.
Mater. Adv..

[5] da
Silva M. D., Moreira R. P. L., Rosa A. P., Borges A. C. (2025). Optimization
of microwave-assisted extraction of tannins and phenolic compounds
obtained from the pequi tree (Caryocar brasiliense). Chem. Eng. Process. Process Intensif..

[6] da
Silva M. D., Martins E. A., Moreira R. P. L., Rosa A. P., Borges A. C. (2025). Valorization of Caryocar brasiliense Byproducts: Microwave-Assisted
Extraction of Phenolics and Material Characterization for Environmental
and Bioenergy Applications. ACS Omega.

[7] Brasil, “Lei no 15.089, de 10 de janeiro de. 2025 https://www.planalto.gov.br/ccivil_03/_ato2023-2026/2025/Lei/L15089.htm. Accessed: Apr. 01, 2025.

[8] Leng L., Xiong Q., Yang L. (2021). An overview on engineering
the surface area and porosity of biochar. Sci.
Total Environ..

[9] Buaki-Sogó M., Zubizarreta L., García-Pellicer M., Quijano-López A. (2020). Sustainable
carbon as efficient support for metal-based nanocatalyst: Applications
in energy harvesting and storage. Molecules.

[10] Cheekatamarla P. (2024). Hydrogen and
the Global Energy Transition—Path to Sustainability and Adoption
across All Economic Sectors. Energies.

[11] Wang, Y. ; Cao, Q. ; Liu, L. A review of low and zero carbon fuel technologies: Achieving ship carbon reduction targets 2022 54 102762 10.1016/j.seta.2022.102762.

[12] Hu X., Xie Q., Zhang J., Yu Q., Liu H., Sun Y. (2020). Experimental
study of the lower flammability limits of H2/O2/CO2 mixture. Int. J. Hydrogen Energy.

[13] Wang C., Wang Q., Fu F., Astruc D. (2020). Hydrogen Generation
upon Nanocatalyzed Hydrolysis of Hydrogen-Rich Boron Derivatives:
Recent Developments. Acc. Chem. Res..

[14] Sperandio G. H., de Carvalho J. P., de Jesus C. B. R. (2024). Hydrogen Evolution from
NaBH4 Using Novel Ni/Pt Nanoparticles Decorated on a Niobium-Based
Composite. Int. J. Hydrogen Energy.

[15] Akbayrak S., Özkar S. (2018). Ammonia borane
as hydrogen storage materials. Int. J. Hydrogen
Energy.

[16] de
Souza N. C. S., Olímpio B. R., Dias G. d. C. (2025). Sustainable hydrogen production *via* NH _3_ BH _3_ hydrolysis using platinum nanoparticles decorated
on solvent-free synthesized LiNbWO _6_. RSC Adv..

[17] Eghbali P., Gürbüz M. U., Ertürk A. S., Metin Ö. (2020). In situ synthesis of dendrimer-encapsulated
palladium(0)
nanoparticles as catalysts for hydrogen production from the methanolysis
of ammonia borane. Int. J. Hydrogen Energy.

[18] dos
Santos J. L., Junior I. M., Sperandio G. H. (2025). Platinum and other Metals Doped-Manganese Tungstate as a Novel Catalytic
Support for NaBH4 Hydrolysis and Hydrogen Evolution Under Mild Conditions. J. Braz. Chem. Soc..

[19] Bousada G. M., da Silva V. N., de Souza B. F. (2024). Niobic acid as a support
for microheterogeneous nanocatalysis of sodium borohydride hydrolysis
under mild conditions. RSC Adv..

[20] da
Silva K. S., de Oliveira Brahim Cortez M., Mazzini L. F. M. (2025). Sustainable Hydrochar from Orange Peel and Bagasse:
A Wet Pyrolysis Approach for Efficient Fe2+ and Mn2+ Removal from
Water Using a Factorial Design. Processes.

[21] Licona–Aguilar A. I., Torres–Huerta A. M., Domínguez–Crespo M. A. (2024). Valorization of agroindustrial
orange peel waste during the optimization
of activated carbon–multiwalled carbon nanotubes–zinc
oxide composites used in the removal of methylene blue in wastewater. Chem. Eng. J..

[22] Silva H. D. M., Alcantara G. U., de Souza L. Z. M. (2023). Production and characterization
of biochar from sugarcane straw. Rev. Mater..

[23] Wu J., Jiang R., Liu S., Zheng G., Liu P., Zheng X. (2024). Desirable performance
and mechanism of RuPd nanoalloys in catalyzing
hydrolytic dehydrogenation of NH3BH3. J. Alloys
Compd..

[24] Junior I. M., Sperandio G. H., Lopes R. P. (2024). Efficient hydrogen evolution from
NaBH4 using bimetallic nanoparticles (Ni–Co) supported on recycled
Zn–C battery electrolyte paste. Int.
J. Hydrogen Energy.

[25] Souza E. I. P., Favero U. G., Sperandio G. H., Andrade T. A., Moreira R. P. L., Hespanhol M. C. (2025). Sustainable
nanocatalyst synthesized from battery waste
for enhanced hydrogen evolution: A circular economy approach. J. Environ. Chem. Eng..

[26] Júnior L. D., De Castro W. C., Bonatto E. C. S., de N. P., Falcão S. (2022). Caracterização
de biocarvões produzidos com resíduos de açaí
e castanha-do-Brasil/Characterization of biochar produced with waste
from açai and Brazil nuts. Braz. J. Dev..

[27] de
Souza N. C. S., do Carmo Dias G., Puiatti G. A. (2025). Eco-Friendly
Photodegradation of Direct Red 80 Dye Mediated by Biochar Decorated
with Cobalt Ferrite. Int. J. Environ. Sci. Technol..

[28] Zhou Y., Xiao X., Li Z., Zhang B., Chen B. (2025). Magnetic zero
valent nickel nanoparticles composite with algal-derived biochar for
superior removal performance toward phenanthrene. Sep. Purif. Technol..

[29] da
Silva M. D., da Boit Martinello K., Knani S. (2022). Pyrolysis
of citrus wastes for the simultaneous production of adsorbents for
Cu­(II), H2, and D-limonene. Waste Manage..

[30] Netto A. M., Nascimento M. C. G. M., de Caux L. S. (2025). Tertiary Treatment of
Pulp Industry Effluents Using Activated Biochar Derived from Biological
Sludge Within a Circular Economy Framework. Processes.

[31] Walbrück K., Kuellmer F., Witzleben S., Guenther K. (2019). Synthesis and characterization
of PVP-stabilized palladium nanoparticles by XRD, SAXS, SP-ICP-MS,
and SEM. J. Nanomater..

[32] Yang G., Hu Q., Hu J. (2023). Hydrogen-rich syngas production from biomass
gasification using biochar-based nanocatalysts. Bioresour. Technol..

[33] Souza T. F., Lobato R. L. M., Maia L. C. (2025). Biochar-Iron Oxide Composites
for Adsorption of Chlorophenoxy Herbicides: Impact of Chlorine Substituents
on Bath and Continuous Adsorption Performance. Adv. Sustainable Syst..

[34] Thommes M., Kaneko K., Neimark A. V. (2015). Physisorption of gases,
with special reference to the evaluation of surface area and pore
size distribution (IUPAC Technical Report). Pure Appl. Chem..

[35] Cortez M. O. B., da Silva K. S., Mazzini L. F. M. (2026). Synthesis and Characterization
of Sustainable Hydrochar from Banana Peels for the Removal of Iron
and Manganese Ions in Aqueous Systems. J. Braz.
Chem. Soc..

[36] Zhang G., Ju P., Lu S. (2024). Efficient adsorption of antibiotics in aqueous
solution through ZnCl2-activated biochar derived from Spartina alterniflora. Colloids Surf., A.

[37] Liu H., Huang M., Tao W., Han L., Zhang J., Zhao Q. (2024). A Palladium Catalyst Supported on
Boron-Doped Porous Carbon for Efficient
Dehydrogenation of Formic Acid. Nanomaterials.

[38] Yameen M. Z., Naqvi S. R., Juchelková D., Khan M. N. A. (2024). Harnessing the
power of functionalized biochar: progress, challenges, and future
perspectives in energy, water treatment, and environmental sustainability. Biochar.

[39] Butenko D. S., Li S., Kotsyubynsky V. O. (2021). Palladium nanoparticles
embedded in microporous carbon as electrocatalysts for water splitting
in alkaline media. Int. J. Hydrogen Energy.

[40] Lopes R. P., Astruc D. (2021). Biochar as a support
for nanocatalysts and other reagents:
Recent advances and applications. Coord. Chem.
Rev..

[41] dos
Santos Silva A. A., Bousada G. M., Mazzini L. F. M. (2024). Biochar from malt residue: Toward a circular economy for sustainable
fluoroquinolone removal in aqueous systems. J. Anal. Appl. Pyrolysis.

[42] Dongre S. S., Zuccante G., Muhyuddin M. (2025). Innovative biochar-based
electrocatalysts from chilli plants and fruits for sustainable oxygen
reduction and hydrogen evolution reactions. Electrochim. Acta.

[43] Zhu C., Zhang J., Huang G., Zhu D. Z. (2024). UV-modified biochar-Bacillus
subtilis composite: An effective method for enhancing Cd­(II) adsorption
from water. Biochem. Eng. J..

[44] Visser E. D., Seroka N. S., Khotseng L. (2023). Catalytic Properties
of Biochar as
Support Material Potential for Direct Methanol Fuel Cell: A Review. ACS Omega.

[45] Ma J., Guo X., Ji X. (2024). Amino-functionalized
CuFe2O4 supported Pd nanoparticles
as magnetically catalyst for H2 production from methanolysis of ammonia
borane and hydrogenation of nitro aromatics. Int. J. Hydrogen Energy.

[46] Choudhary M., Singh S., Sinha A. K. (2024). Enhanced hydrogen gas
sensing using palladium – graphene oxide (PdGO) thin films. Chem. Eng. J..

[47] Liu Q., Ran W., Bao W., Li Y. (2025). A Review on Catalytic Hydrolysis
of Ammonia Borane for Hydrogen Production. Energies.

[48] Long J., Wu H., Liu Y. (2024). Hydrogen production from chemical hydrogen
storage materials over copper-based catalysts. cMat.

[49] Sun Q., Zhang H., Fan Y., Bian L., Peng Q., Liu B. (2023). Regulating the electronic
structure of Pd nanoparticles through metal
alloy–support interactions for enhanced hydrogen generation. Renewable Energy.

[50] Wang W., Lu Z., Luo Y., Zou A., Yao Q., Chen X. (2018). Mesoporous
Carbon Nitride Supported Pd and Pd-Ni Nanoparticles as Highly Efficient
Catalyst for Catalytic Hydrolysis of NH_3_BH_3_. ChemCatChem.

[51] Zhang L., Lin H., Liu Q., Xu M., Hu C., Zhang H. (2025). B-doped Co3O4–Pd
nanocomposites on halloysite nanotubes for enhanced hydrogen generation
via ammonia borane hydrolysis. Int. J. Hydrogen
Energy.

[52] Liu S., Chen X., Wu Z.-J., Zheng X.-C., Peng Z.-K., Liu P. (2019). Chitosan-reduced graphene
oxide hybrids encapsulated Pd(0) nanocatalysts
for H2 generation from ammonia borane. Int.
J. Hydrogen Energy.

[53] Cao Y., Yang S., Liu P., Zhu Q., Zheng X. (2025). Nickel-promoted
ruthenium nanocatalysts for controllable hydrogen production from
NH3BH3 hydrolysis. Appl. Surf. Sci..

[54] Sperandio G. H., de Carvalho J. P., de Jesus C. B. R. (2024). Hydrogen evolution from
NaBH4 using novel Ni/Pt nanoparticles decorated on a niobium-based
composite. Int. J. Hydrogen Energy.

[55] Deng J., Zhou X., Zou J., Qin Y., Wang P. (2022). “PdCo
Alloy Supported on a ZIF-Derived N-Doped Carbon Hollow Polyhedron
for Dehydrogenation of Ammonia Borane. ACS Appl.
Energy Mater..

[56] Zhao H., Liu Y., Zhang W. (2024). NiPdMo Nanoparticles Reduced by Cs­[closo-B6H7]
as High-Performance Catalysts for Hydrogen Generation from Hydrolysis
of Ammonia Borane. Int. J. Hydrogen Energy.

[57] Twombly A. T., Harris J. W. (2025). Kinetics of hydrogen
release from ammonia borane and
role of the support for supported Ru catalysts in methanol solvent. J. Catal..

[58] Yang X., Wei J., Wang Q. (2020). Pd–Ru nanocatalysts derived from a Pd-induced
aerogel for dramatic boosting of hydrogen release. Nanoscale.

[59] Tonbul Y., Akbayrak S., Özkar S. (2016). Palladium­(0) Nanoparticles Supported
on Ceria: Highly Active and Reusable Catalyst in Hydrogen Generation
from the Hydrolysis of Ammonia Borane. Int.
J. Hydrogen Energy.

[60] Zhao X., Hu H., Li G., Cai J., Wang Y., Fan G. (2022). Low-temperature
control over deposition of ultrafine Pd nanoparticles on porous carbon
nanosheets for highly efficient dehydrogenation of ammonia borane. J. Alloys Compd..

[61] Zhao H., Liu Y., Zhang W. (2024). NiPdMo nanoparticles reduced by Cs­[closo-B6H7]
as high-performance catalysts for hydrogen generation from hydrolysis
of ammonia borane. Int. J. Hydrogen Energy.

[62] Karataş Y., Zengin A., Gülcan M. (2022). Preparation
and characterization
of amine-terminated delafossite type oxide, CuMnO2–NH2, supported
Pd (0) nanoparticles for the H2 generation from the methanolysis of
ammonia-borane. Int. J. Hydrogen Energy.

[63] Mao M., Chen Q., Wu J., Fan G. (2020). Anchoring and space-confinement
effects to synthesize ultrasmall Pd nanoparticles for efficient ammonia
borane hydrolysis. Int. J. Hydrogen Energy.

[64] Akbayrak S., Çakmak G., Öztürk T., Özkar S. (2021). Rhodium­(0),
Ruthenium­(0) and Palladium(0) nanoparticles supported on carbon-coated
iron: Magnetically isolable and reusable catalysts for hydrolytic
dehydrogenation of ammonia borane. Int. J. Hydrogen
Energy.

[65] Liu S., Li Y.-T., Zheng X.-C., Guan X.-X., Zhang X.-L., Liu P. (2020). Pd nanoparticles
anchoring to core-shell Fe3O4@SiO2-porous carbon
catalysts for ammonia borane hydrolysis. Int.
J. Hydrogen Energy.

[66] Zhou Y.-H., Cao X., Ning J., Ji C., Cheng Y., Gu J. (2020). Pd-doped Cu
nanoparticles confined by ZIF-67@ZIF-8 for efficient dehydrogenation
of ammonia borane. Int. J. Hydrogen Energy.

[67] Deka J. R., Saikia D., Chen P.-H., Chen K.-T., Kao H.-M., Yang Y.-C. (2020). Palladium nanoparticles
encapsulated in carboxylic
acid functionalized periodic mesoporous organosilicas as efficient
and reusable heterogeneous catalysts for hydrogen generation from
ammonia borane. Mater. Res. Bull..

[68] Tonbul Y., Akbayrak S., Özkar S. (2016). Palladium­(0)
nanoparticles supported
on ceria: Highly active and reusable catalyst in hydrogen generation
from the hydrolysis of ammonia borane. Int.
J. Hydrogen Energy.

