# Supplementary material for: Pequi Seed Biochar as Pd Nanoparticle Support for Catalytic Hydrogen Evolution from Ammonia Borane
Source: ACS Omega. 2026 Jun 30;11(27):39775–87. doi: 10.1021/acsomega.5c13606 (PMC13382663; doi:10.1021/acsomega.5c13606)
Supplement: Supplementary file 1 [file ao5c13606_si_001.pdf]

Supporting Information

Pequi Seed Biochar as Pd Nanoparticle Support for Catalytic Hydrogen Evolution from Ammonia Borane

Júlia Araújo Lavorato[a], Marcela de Oliveira Brahim Cortez[a], Mariele Dalmolin da Silva[b], Antonio Machado Netto[a], Lavínia Nunes Louzada[a], Noemi Cristina Silva de Souza[a], Leonarde do Nascimento Rodrigues[c], Renê Chagas da Silva[c], Luciano de Moura Guimarães[c], Didier Astruc[d] and Renata Pereira Lopes Moreira[a*].

[a]Departament of Chemistry, Universidade Federal de Viçosa (UFV). Av. Peter Henry Rolfs, s/n, Campus Universitário. Viçosa – MG, Brazil. CEP: 36570-900.

[b]Departament of Agricultural Engineering, Universidade Federal de Viçosa (UFV). Av. Peter Henry Rolfs, s/n, Campus Universitário. Viçosa – MG, Brazil. CEP: 36570-900.

[c]Department of Physics, Universidade Federal de Viçosa (UFV). Av. Peter Henry Rolfs, s/n, University Campus. Viçosa – MG, Brazil. CEP: 36570-900.Viçosa – MG, Brazil. CEP: 36570-900.

[d]ISM, UMR CNRS N° 5255, University of Bordeaux, Talence Cedex, 33405, France.

* renata.plopes@ufv.br (Corresponding Author)

Table of Contents

Figure S1. Experimental setup used in the reactions: (1) thermostatic bath for temperature control; (2) Schlenk tube; (3) rubber septum for sealing; (4) thermometer; (5) connection tubing; (6) burette; and (7) system open to the atmosphere 3

Figure S2. Raman spectroscopy of biochar (BCZ) 3

Figure S3. N_2 Adsorption–Desorption Isotherm of (a) biochar without $ZnCl_2$, (b) biochar with ZnCl2 (BCZ) and (c) metal nanoparticles support in biochar (Pd NPs-BCZ)................4

Figure S4. X-ray Diffraction (XRD) of (a) biochar (BC) and (b) Pd NPs-BCZ catalyst 5

Figure S5. Zeta Potential of biochar (BCZ) 5

Figure S6. Energy dispersive spectroscopy (EDS) spectrum for Pd NPs-BCZ 6

Figure S7. Selected-area electron diffraction (SAED) pattern of Pd NPs–BCZ....................6

Figure S8. Evaluation of catalyst variants for hydrogen evolution from NH3BH3. (a) TOF values; (b) kinetic relationships. Reaction conditions: 2 mmol% metal, 20 mg BCZ, 0.58 mmol L-1 of NH3BH3 and 293.15 K 7

Figure S9. Transmission electron microscopy (TEM) of Pd NPs-BCZ after 20 successive reuse cycles. 7

Table S1. ICP analysis of Pd, B, and Zn contents in the materials before and after H_2 evolution experiments. 8

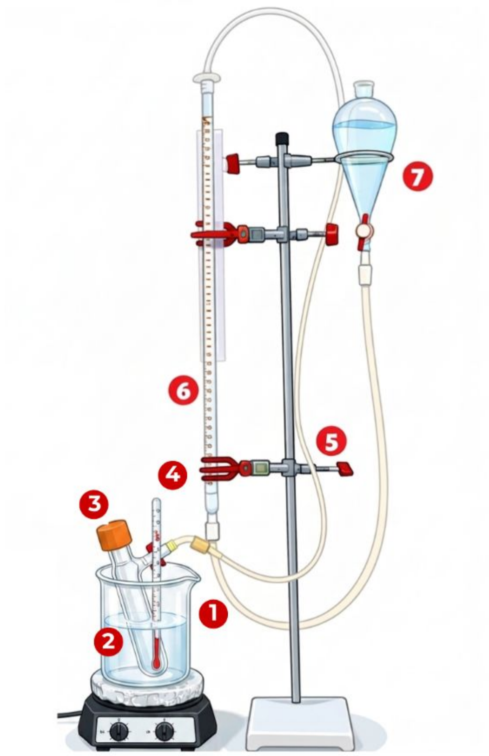

Figure S1. Experimental setup used in the reactions: (1) thermostatic bath for temperature control; (2) Schlenk tube; (3) rubber septum for sealing; (4) thermometer; (5) connection tubing; (6) burette; and (7) system open to the atmosphere

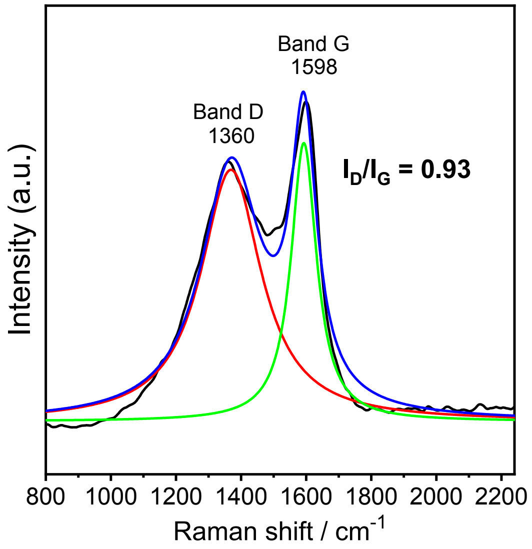

Figure S2. Raman spectroscopy of biochar (BCZ)

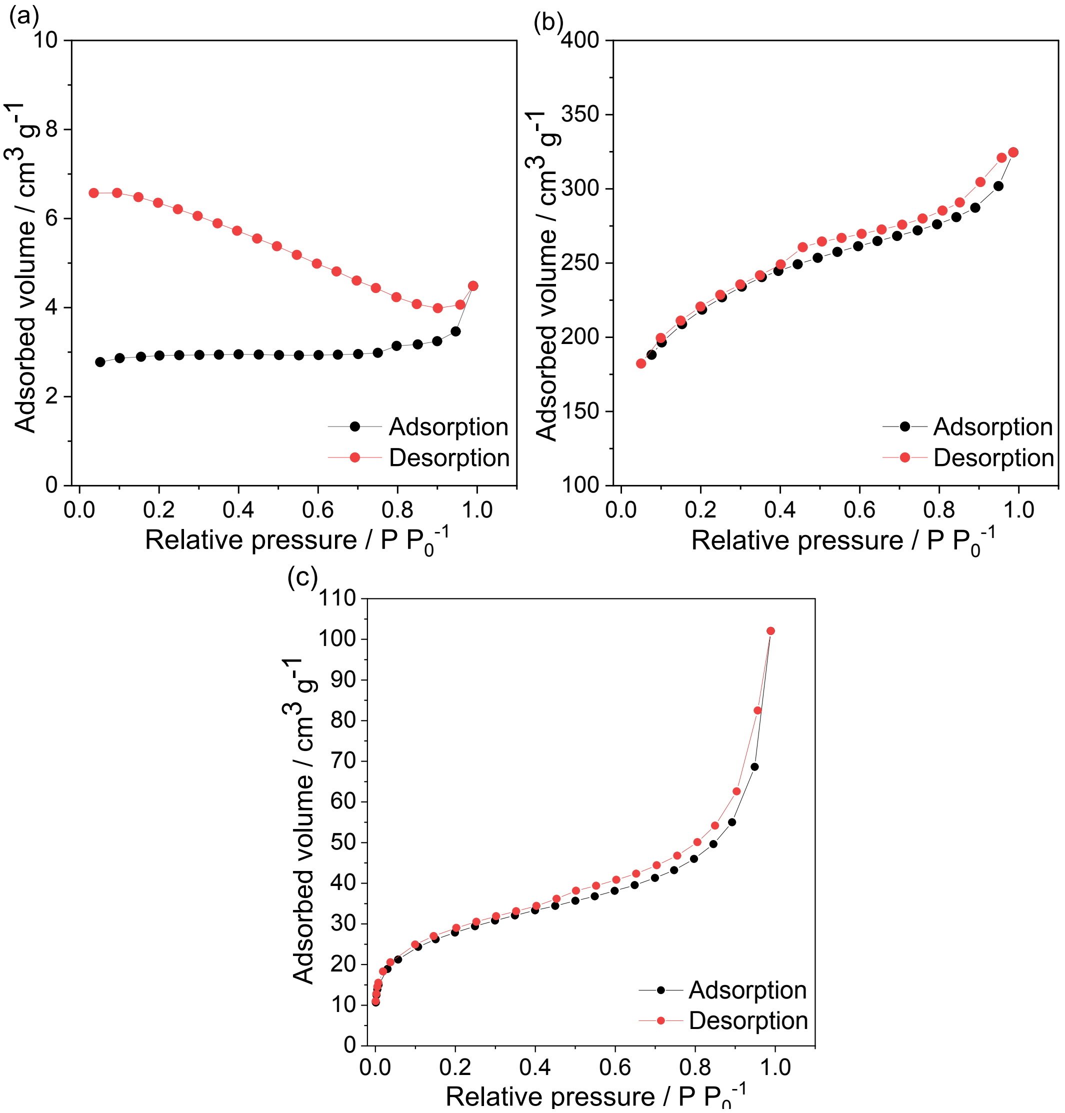

Figure S3. N_2 Adsorption–Desorption Isotherm of (a) biochar without $ZnCl_2$, (b) biochar with ZnCl2 (BCZ) and (c) metal nanoparticles support in biochar (Pd NPs-BCZ)

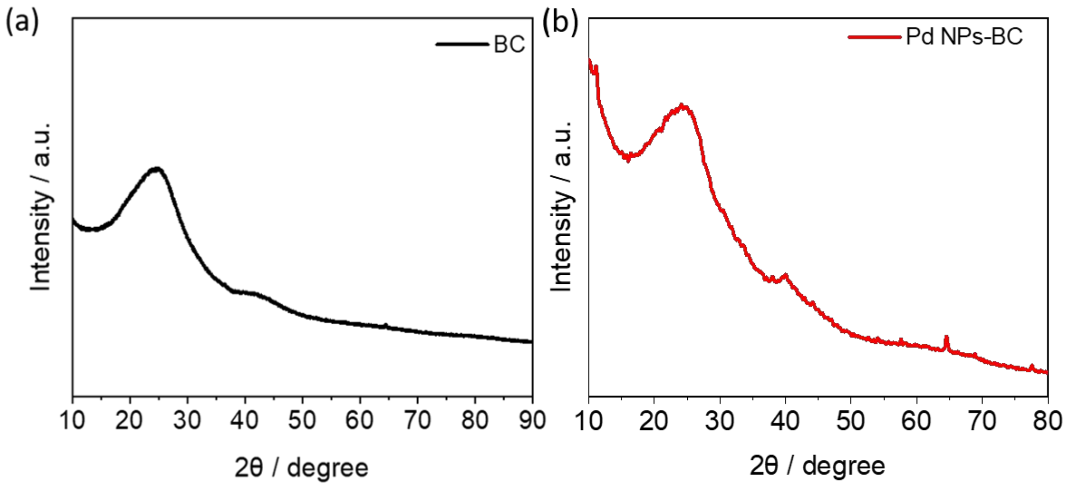

Figure S4. X-ray Diffraction (XRD) of (a) biochar (BC) and (b) Pd NPs-BCZ catalyst

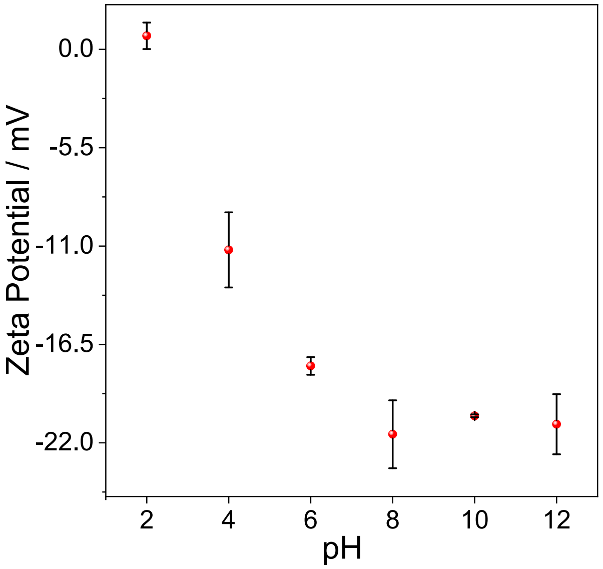

Figure S5. Zeta Potential of biochar (BCZ)

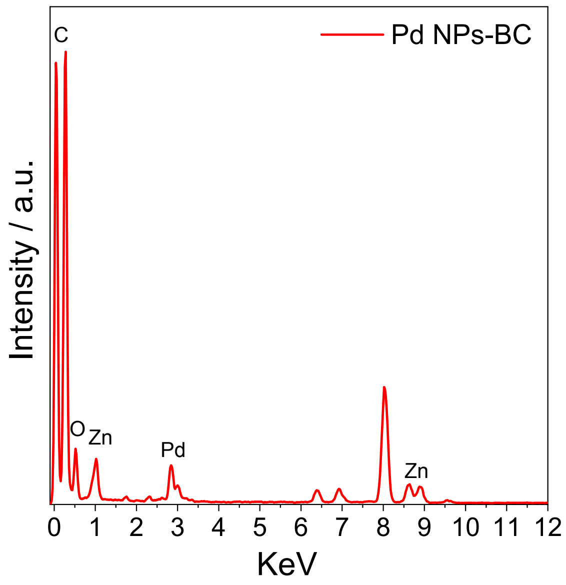

Figure S6. Energy dispersive spectroscopy (EDS) spectrum for Pd NPs-BCZ

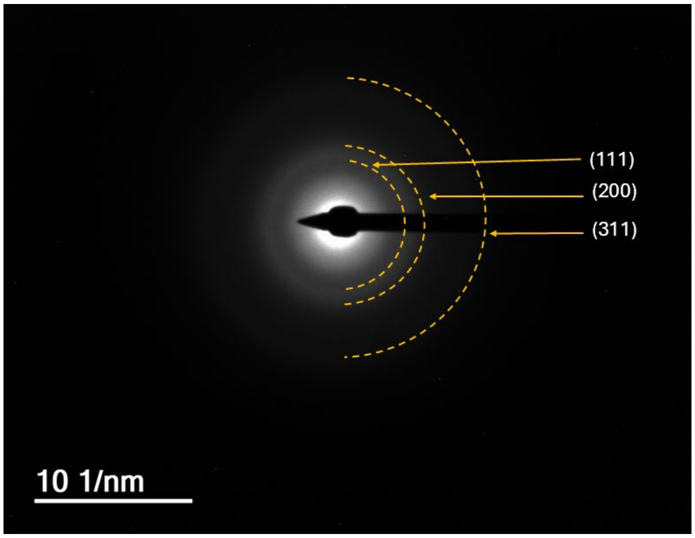

Figure S7. Selected-area electron diffraction (SAED) pattern of Pd NPs–BCZ

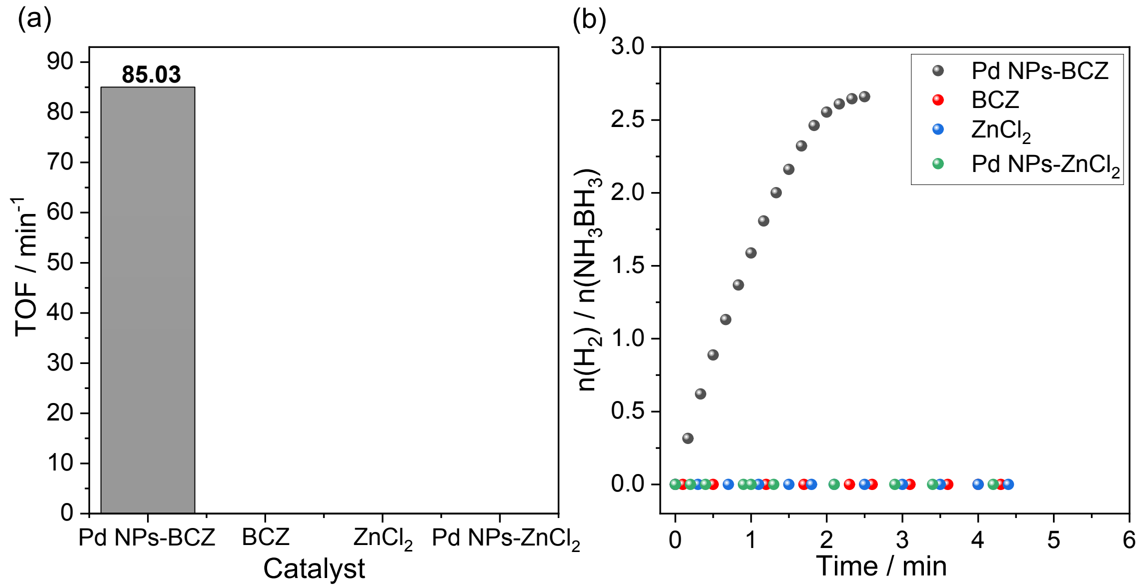

Figure S8. Evaluation of catalyst variants for hydrogen evolution from NH3BH3. (a) TOF values; (b) kinetic relationships. Reaction conditions: 2 mmol% metal, 20 mg BCZ, 0.58 mmol L-1 of NH3BH3 and 293.15 K

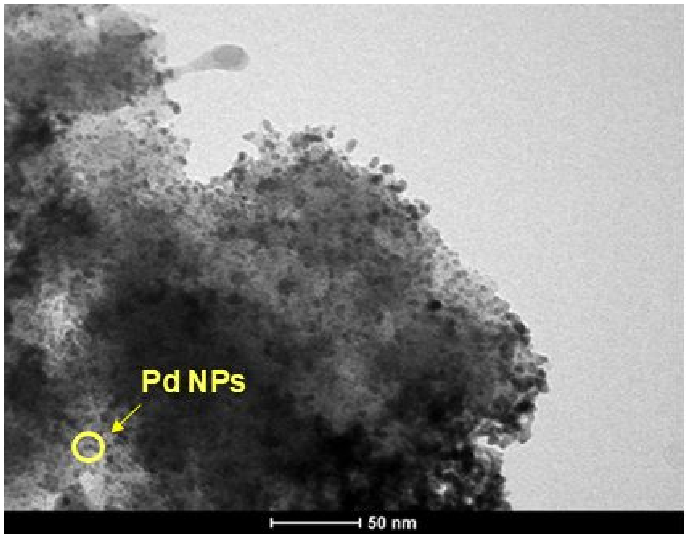

Figure S9. Transmission electron microscopy (TEM) of Pd NPs-BCZ after 20 successive reuse cycles.

Table S1. ICP analysis of Pd, B, and Zn contents in the materials before and after H_2 evolution experiments.

Catalyst	Mass of Pd (μmol)	Mass of B (μmol)	Mass of Zn (μmol)
**Fresh (as-prepared catalyst)**	10.2 ± 2.3	18.1 ± 0.9	25.8 ± 0.3
**Reused (after H_2 evolution cycles)**	10.1 ± 2.3	578.7 ± 0.9	17.3 ± 0.3
